# Effects of *Tithonia diversifolia* Extract as a Feed Additive on Digestibility and Performance of Hair Lambs

**DOI:** 10.3390/ani14243648

**Published:** 2024-12-17

**Authors:** Olga Teresa Barreto-Cruz, Juan Carlos Henao Zambrano, Maria Alejandra Ospina Barrero, Román David Castañeda-Serrano

**Affiliations:** 1 Laboratory of Animal Nutrition, Veterinary Medicine and Animal Science Program, Universidad Cooperativa de Colombia, Ibagué 730003, Tolima, Colombia; 2 Faculty of Veterinary Medicine and Animal Science, Universidad del Tolima, Ibagué 730006, Tolima, Colombia

**Keywords:** apigenin, additives, caffeic acid, fibre digestion, phyto-additives, luteolin, quercetin

## Abstract

Animal production requires efficiency, safety and environmental sustainability. Bioactive compounds from tropical plants could modulate ruminal fermentation, providing an alternative method to antibiotic treatment and addressing concerns about antibiotic resistance. In this study, the aim was to determine the effects of *Tithonia diversifolia* extract (TDE) on performance, intake, digestibility and blood parameters [i.e., glucose, blood urea nitrogen (BUN), aspartate aminotransferase (AST), alanine aminotransferase (ALT)] in crossbreed sheep. The main biocompounds of the TDE include caffeic acid (CA), quercetin (QCT), luteolin (LT) and apigenin (AP). Experiment 1: An in vitro dry matter digestibility (IVDMD) study was conducted to determine the optimal inclusion levels. The IVDM values were 73.09a, 82.03b, 81.01b, 73.20a and 74.51a for the control, 5, 10, 15 and 20 g/kg for the DM treatments, respectively (R-Sq adj = 0.857). The levels of 5 and 10 g were selected for the in vivo experiment. Experiment 2: Twenty-eight male crossbred hair lambs were assigned to four treatments (n = 7): control, 20 mg monensin/day, 5 g TDE/day and 10 g TDE/day groups. No differences in animal performance were observed, including body weight and feed conversion (*p* > 0.05). The TDE at 10 g/day improved NDF digestibility) (61.32%) and reduced the ruminal acetate to propionate ratio. The total digestible nutrients (TDN) were higher in 10 g TDE treatment with 66.41% and the lowest acetate production (67.82%) (*p* = 0.042), and propionate production (21.07%) were observed. The TDE were safe at 5 g and 10 g/day for liver function and exhibited lower BUN levels suggesting an improvement in protein metabolism. TDE extract at 10 g/day (TDE10), showed improvements in total tract digestibility of NDF and reduced the ruminal acetate to propionate ratio. However, due to TDE10 reducing the DM intake, the improvements in digestibility and ruminal fermentation were not reflected in growth performance improvements.

## Simple Summary

In this study, the aim was to investigate the potential of polyphenol mixtures as natural modulators of ruminal fermentation, which can serve as an alternative to antibiotics in animal production. The findings of this study suggested that biocompounds from tropical plants can improve ruminant production efficiency, while reducing reliance on antibiotics and addressing concerns about livestock efficiency and safety.

## 1. Introduction

The use of biocompounds as alternatives to antibiotics for modulating ruminal fermentation [1] has become a key research area for reducing antibiotic resistance strains [2,3,4] and developing sustainable livestock systems that ensure feed efficiency, maintain low methane emissions and produce safe animal products [5].

Replacing synthetic antibiotics with secondary biocompounds of tropical plants could help to address concerns over antibiotic resistance while maintaining productivity and efficiency [6].

Antibiotics, such as monensin, exhibit their antimicrobial effects through a single mechanism [7]. In contrast, polyphenols synthesised by plants in response to biotic or abiotic factors [8] could influence ruminal fermentation [9,10,11] via diverse pathways damaging cytoplasmic membranes, inhibiting nucleic acid synthesis, disrupting energy metabolism and blocking cell wall and membrane synthesis [12,13]. These effects primarily target Gram-positive bacteria, which are associated with increased acetate production at the ruminal level. By differentially regulating these bacterial populations, propionate production can be enhanced in the rumen [14]. This alteration in volatile fatty acids (VFA) improves energy utilisation and overall efficiency in ruminants.

The effects of polyphenols as pure compounds have been studied [15,16,17]. Quercetin (QCT) has shown potential benefits, increased gas production and suppressed methane production without negatively impacting ruminal microbial fermentation in vitro [18]. An in vitro study [17] tested QCT and luteolin-7-glucoside at dosages of 0.5, 5.0 and 50.0 mg/g and found that the higher dosages effectively reduced methane production, and luteolin-7-glucoside, similar to tannic acid, also lowered the ammonia concentrations. These findings suggest that certain polyphenols could mitigate methane and ammonia formation. However, further research on live animals is needed to confirm this.

The synergistic interactions between different bioactive compounds could be an important pathway to achieve the desired effects on the rumen ecosystem at low doses [19,20,21].

The secondary biocompounds of *Tithonia diversifolia* (Hemsl.) A. Gray, primarily polyphenols, have demonstrated antimicrobial, antiparasitic and antiprotozoal effects [22,23]. Moreover, a reduction in methane production was observed in vitro when forage was included [24,25]. Further research is needed to evaluate the potential effects of *Tithonia diversifolia* extracts (TDE) and their biocompounds on certain parameters, such as feed intake, digestibility, VFA production, and animal performance.

Conversely, it is necessary to identify the effects of these extracts on the liver and kidneys in vivo to determine their innocuous effects because these organs metabolise the biocompounds. The blood urea nitrogen (BUN) measures kidney function and protein metabolism [26], whereas the aspartate aminotransferase (AST) and alanine aminotransferase (ALT) is involved in liver function and metabolism [27]. Moreover, the glucose levels provide further insights into energetic metabolism [28].

In this study, the aim was to determine the effects of *Tithonia diversifolia* extract (TDE) on performance, intake, digestibility and blood metabolites in crossbreed hair lambs.

## 2. Materials and Methods

Study Location and Animal Care. This study was conducted at an altitude of 1168 m above sea level (4°25′59″ N, 75°13′1″ W). 

Plant Material. The *Tithonia diversifolia* (Hemsl.) A. Grey, commonly known as Mexican sunflower (Asteraceae: Heliantheae) or false sunflower is a shrub perennial [29]. Moreover, it is a highly invasive species that has become naturalized in regions such as Africa, Australia and Asia [30]. The *T. diversifolia* leaves were collected 90 days after pruning at the Nataima Research Centre—Agrosavia, Colombia at 431m above sea level, with an average temperature of 28 °C, annual precipitation of 1474mm and 70% relative humidity. Leaves were dried at 40 °C for 72 h, ground, vacuum-packed and stored at 4 °C pre-extraction.

Extract preparation. The TDE was obtained from powdered dried leaves and distilled water (1:10, *w*/*v*) by stirring for 24 h/160 rpm. The obtained extract was filtered through common filter paper and the TDE was stored at 0 °C until use. 

Phytochemical analysis and quantification of the major compounds. An HPLC analysis was performed using the UHPLC Dionex™ UltiMate™ 3000; Sunnyvale, CA, USA coupled to an Orbitrap™ mass detector (Exactive Plus), with a heated-electrospray interface (HESI-II) in positive ion mode (350 °C). Separation was achieved using a Hypersil GOLD™ aQ column; Waltham, MA, USA (100 mm × 2.1 mm id, × 1.9 μm particle size). The mobile phase consisted of (A) 0.1% *v*/*v* formic acid and 5 mM ammonium formate (water), and (B) 0.1% *v*/*v* formic acid and 5 mM ammonium formate (methanol). Moreover, the initial gradient condition transitioned from 100% A to 100% B over 8 min, followed by 4 min maintained at 100% B, and then returned to 100% A within 1 min. The data analysis was performed using the Thermo XCalibur™, Waltham, MA, USA Roadmap software (version 3.1.66.10). Compound identification relied on extracted ion current, exact masses of protonated target compounds and comparison with certified standards. The quantification of the major compounds in the TDE revealed that each gram contained 0.0498 mg of caffeic acid (CA), 0.001 mg of quercetin (QCT), 0.0007 mg of luteolin (LT) and 0.0003 mg of apigenin (AP) (Table 1).

**Figure 1:**
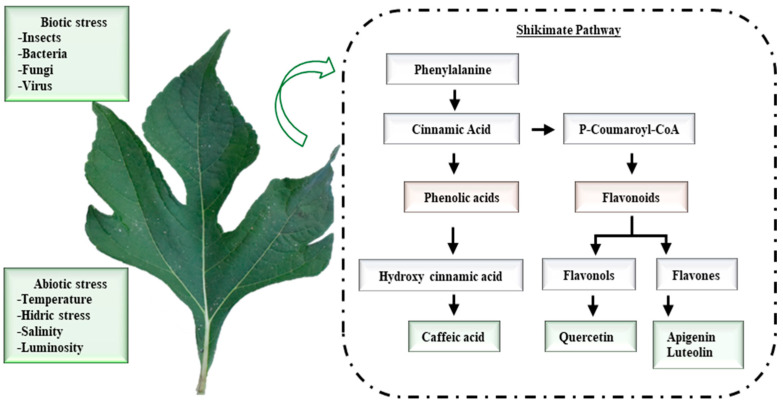
Biosynthesis of the main polyphenols found in leaves of aqueous extracts of *Tithonia diversifolia*.

These polyphenols (Figure 1) are products of shikimic acid metabolism, with phenylalanine [9] as a common precursor.

Experiment 1. In vitro.

Before the in vivo experiment, the digestibility of dry matter (IVDMD) protocol was performed to determine the inclusion levels of TDE. It followed the guidelines for the DAISYII^®^ incubator (ANKOM Technology, Fairport, NY, USA), using Ankom FN° 57 bags with 0.5 g of sample per bag. For each treatment, the bags were conditioned in 2000 mL glass jars, including one blank bag (empty and sealed), for the correction factor for potential particle ingress or weight loss of the bags. The rumen inoculum was obtained using a vacuum pump and transported with CO_2_ until incubation. Moreover, this was collected from a cannulated bovine feeder with the same diet proportions to obtain a microbiota ratio similar to the experimental conditions (21 days before collection). The bags were incubated for 48 h at 39.2 ± 0.5 °C. The doses were selected based on preliminary tests conducted with different levels of in vitro inclusion to establish relevant usage doses for this study. Four levels of TDE inclusion were evaluated and distributed across the following five treatments: control (without additives), 5 g per kg of incubated DM, 10 g per kg of incubated DM, 15 g per kg of incubated DM and 20 g per kg of incubated DM. Moreover, the experiment was repeated two times, with four replicates in each experiment. These dosages were equivalent to the amount of treatment applied to each jar (2000) mL containing 31.25, 62.5, 125, 250 and 375 mg/kg of DM. The diet had a corn silage and concentrate ratio of 60:40 (corn, soybean meal, and mineral supplement).

Experiment 2. In vivo experiment.

Animals and diet. Twenty-eight intact male crossbred hair lambs (Katahdin, Santa Ines and Pelibuey), with a mean body weight of 18.52 ± 4.57 kg and average age of 3 months, were assigned to the following four treatments (n = 7 animals): control (no additives), 20 mg monensin, 5.0 g (TD5) and 10 g (TD10) TDE/day groups. For the TDE treatments, the final amounts of the polyphenols were 0.249 mg CA, 0.005 mg QCT and 0.0035 mg LT and 0.0015 mg AP for TD5, and 0.498 mg CA, 0.01 mg QCT and 0.007 mg LT and 0.003 AP for TD10. Moreover, the animals were housed in individual pens for 57 days and fed twice daily with the same diet. The first 15 days were for adaptation to the pens, allowing them to become adapted to individual confinement since they were transitioning from free-range grazing, followed by 42 days of experimental supplementation with the treatments. The diet (Table 2) consisted of a total mixed ratio based on corn silage with a 66:34 ratio (twice a day: at 8:00 a.m. and 4:00 p.m., with an allowance of 5%–10% leftovers with ad libitum access to water. The diet was formulated according to NRC requirements [31].

Intake and digestibility. Animals were fed with the experimental diets for 42 days; samples were taken for analysis during the last four consecutive days. Daily intake was monitored throughout the experiment to adjust intake, and during collection days, all provided feed and leftover orts were collected. Individual samples were taken from each animal every four days, the samples where dried and then these composed samples were analysed for each animal.

Total faecal production was obtained, as the animals were housed in individual elevated confinements equipped with trays to collect all faecal output. Feed, leftovers and faeces were stored at −20 °C. These samples were processed by pre-drying in a forced ventilation oven at 55 °C for 72 h and then ground to a particle size of 1 mm for chemical analysis. The chemical composition was determined using the methods of AOAC [32] in dry matter (DM) number 930.15, ash number 942.05, crude protein (CP) number 992.15 and ether extract (EE) number 920.39. The neutral detergent fibre (NDF) was determined according to Van Soest [33] along with the modification of Mertens [34]. The non-structural carbohydrates (NSC) were calculated using the formula: NSC = 100 − (NDF + protein + fat + ash) [33]. The total digestible nutrients (TDN) were calculated using equations [35].

Animal performance. The animal performance was determined by the difference between the initial body weight and the final body weight divided by the experimental days. This was calculated using the initial and final body weights measured at the beginning and end of the experiment, starting with the supplementation of TDE on day 1 (following the 15 days of adaptation to the individual pens) until day 42 of supplementation. For the feed conversion (FC), intake was monitored to adjust daily intake and samples for dry matter were collected, using data collected over the last four days. FC was calculated based on intake and animal performance, using the following equation: FC = (DMI/ADG), where DMI is the daily dry matter intake in kg and ADG is the average daily gain in kg.

Volatile fatty acids (VFA). Ruminal fluid was collected after the slaughter of the animals. The samples of 50 mL were acidified with H_2_SO_4_ solution (0.036 N) and frozen at −20 °C for later determination. Reverse phase high-performance liquid chromatography with diode array detector (RP HPLC-DAD) was applied. A liquid chromatograph Agilent Technologies 1260 Series (Palo Alto, CA, USA) equipped with a diode array detector (DAD) set at 210 nm was used. The column used was a Kinetex C18 (100 mm × 4.6 mm × 2.6 μm). Furthermore, acetic acid (Dr. Ehrenstorfer, Lot: G979843, 99.55%), propionic acid (Sigma-Aldrich, Lot: 506071, 99.5%), butyric acid (Sigma-Aldrich, Lot: SHBC1023V, 99.0%) and valeric acid (Sigma-Aldrich, Lot: MKBJ8913V, 99.0%) (Burlington, VT, USA) were used as the reference materials.

Blood Parameters. Samples of blood were taken on day 42 at the end of the experiment. The AST and ALT measurements were taken by enzymatic test [36]. Blood urea nitrogen (BUN) and glucose were measured by colourimetric reactions quantified by spectrophotometry (BioSystems^®^ spectrophotometer, Applied Biosystems, Foster City, CA, USA) [37].

Statistical design. For in vitro digestibility, the effects of the inclusion levels of TDE were analyzed using polynomial regression model; the model was represented as Y = β_0_ + β1_X1_ + β2_X2_ + β3_X3_ + β3_X4_ + β3_X5_, where *Y* represents the predicted in vitro digestibility, *X* denotes the treatment and *β* are the polynomial coefficients determined from the data. For the in vivo experiment, a general linear model was employed, where treatments were considered fixed effects, while the effects of animals were treated as random. The model was represented as Yijk = μ + Ti + Ak + eijk, where Yik represents the observed response for the *k* animal under the *i* treatment, *μ* is the overall mean, *Ti* is the effect of the *i* treatment and e*ik* is the error term. The effects of treatment interactions were evaluated using Fisher’s test (*p* < 0.05). The analyses were performed using Minitab 17 software [38].

## 3. Results

**Figure 2:**
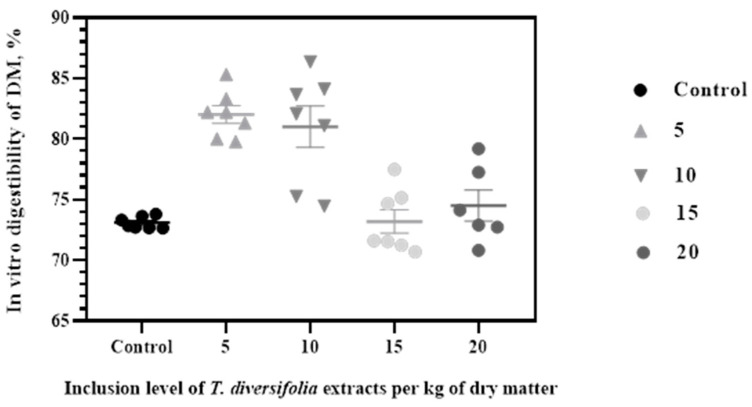
In vitro digestibility of dry matter (IVDMD) in a diet with a forage to concentrate ratio of 60:40 at different inclusion levels of TDE.

For experiment 1, the inclusion of TDE positively affected the IVDMD at low levels (*p* = 0.001). The IVDMD values were 73.09a, 82.03b, 81.01b, 73.20a and 74.51a, for the control (cero), 5, 10, 15 and 20 g/kg of TDE treatments, respectively. The treatments with 5 and 10 g/kg DM showed the highest IVDMD values *p*(=0.001) (Figure 2).

**Figure 3:**
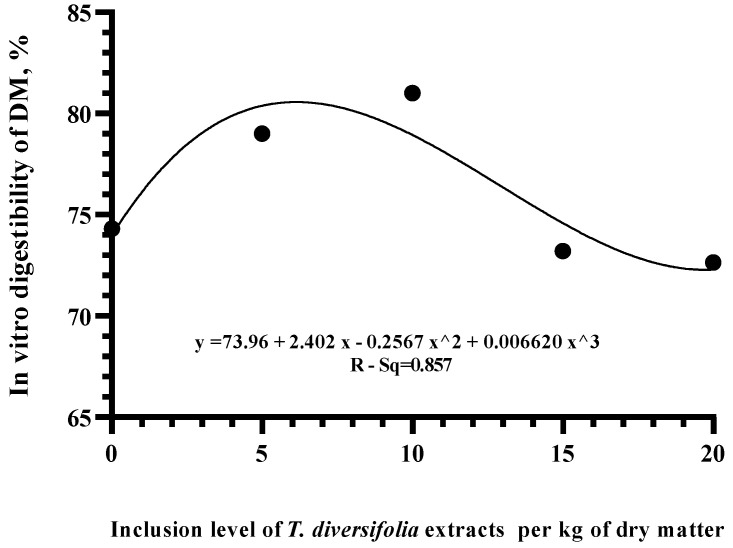
Polynomial regression of in vitro digestibility of dry matter (IVDMD) in a diet with forage to concentrate ratio of 66:34 at different inclusion levels of TDE.

A polynomial regression analysis was performed obtaining a quadratic effect (R-Sq adj = 0.857) on IVDMD with levels about levels above 20 g of TDE (Figure 3). Based on these results, the levels of 5 and 10 g TDE were selected for the in vivo study.

Table 3 shows the animal performance, DM conversion and feed intake average. In relation to animal performance, no significant difference was observed between the treatments for the initial BW (*p* = 0.299), final BW (*p* = 0.075), ADG (*p* = 0.223) and DM conversion (*p* = 0.952). Moreover, no statistical differences were observed for the inclusion levels of TDE.

For the feed intake between treatments, statistical differences were observed (*p* = 0.000) indicating the highest values for the TD5 group, in the DMI (0.90 kg DM/day), the CP intake (0.15 kg/day), the NDF intake (0.37 kg/day) and non-structural carbohydrate (NSC) intake (0.36 kg/day).

Table 4 shows the effects on nutrient digestibility and VFA production. No significant differences were observed between the treatments for digestibility of DM (*p* = 0.849), CP (*p* = 0.676) and NSC (*p* = 0.082). The highest NDF digestibility was observed in the TD10 group (61.32) (*p* = 0.025). For TDN, the TD10 group had the highest TDN (66.41) (*p* = 0.048).

For the VFA, the TD10 group had the lowest acetate percentage (67.82) (*p* = 0.042) and a trend towards a higher percentage of propionate was observed (21.07) (*p* = 0.056). No statistical differences were observed between the butyrate (*p* = 0.147) and acetate/propionate ratio (*p* = 0.106). 

In the present study, no effects on the blood parameters were observed between treatments (*p* > 0.05) (Table 5). However, the BUN (10.93 mg/dL) and AST (10.93 mg/dL) levels were observed to be numerically lower at the TD10.

## 4. Discussion

The TDE significantly enhanced the IVDMD at inclusion levels of 5 g and 10 g/kg of DM. For experiment 1, the inclusion of TDE positively influenced the IVDMD at low levels (*p* = 0.001).

In our previous studies, our findings have suggested that the secondary biocompounds from tropical plants can modulate ruminal fermentation at low doses [19], exhibiting a synergistic effect depending on the type of diet (forage/concentrate ratio) [39,40,41].

These findings are consistent with [16], who investigated the effects of CA (purity of >98.5%; Sigma-Aldrich Co.) in an IVDMD study using high forage and high concentrate ratios (corn silage to concentrate ratio of 75:25 or 25:75). They found that increasing CA levels (0, 20 and 40 g/kg DM) linearly reduced (*p* < 0.05) the digestibility of DM, NDF and total VFA production in the high-forage treatment, while in the high-concentrate treatment, CA had the opposite effect (*p* < 0.05).

Similarly, ref. [42] investigated the effects of polyphenols in different sorghum stalks varieties on total gas production in vitro and found that low levels of caffeic acid (270.64 ng/g) increased propionic acid production (17.12 mmol/L) and improved the acetic to propionic acid ratio (3.88). Furthermore, low levels of apigenin (20.462.63 ng/g) improved antioxidant activity and fermentation efficiency. These findings align with the results of our study, where levels higher than 10 g of TDE (0.498 mg CA, 0.01 mg QCT and 0.007 mg LT and 0.003 AP) reduced IVDMD, demonstrating that the synergistic effects of polyphenols at lower doses optimized ruminal fermentation. Further metagenomic studies are needed to understand the effects of TDE on ruminal bacterial populations at long term supplementation.

For experiment 2, in vivo, in the group control and TDE at 5 g/day an increased DM intake was observed, and the monensin and TDE at 10 g/day group showed a lower intake of DM. The studies indicate that the effects of monensin on ruminal bacteria are time-dependent. For instance, ref. [43] noted that the methane-depressing effects of monensin decreased with prolonged exposure, and the degradation of alfalfa NDF significantly diminished by day 50. 

The results indicated that TDE at 10 g/day improved dry matter intake at the same level as monensin. TDE at 10 g/day improved neutral detergent fibre (NDF) digestibility and altered ruminal fermentation by reducing acetate and potentially increasing propionate levels. This suggests that TDE may modify ruminal fermentation, favouring propionate production, which may reflect changes in microbial populations or metabolic pathways that overall improve the TDN. 

Similar findings were reported in a study on water buffaloes with rumen cannulas, using a 4 × 4 Latin square design with a high-forage diet (corn silage to concentrate ratio of 80:20). The phenolic compounds extracted from honeybee propolis were administered in four doses (00, 16.95, 33.9 and 50.85 mg/d), containing varying amounts of CA (0, 4.81, 9.61 or 14.42 mg/d) and AP (0, 1.29, 2.59 and 3.88 mg/d). Although these phenolic compounds did not impact the overall digestibility, they showed a quadratic increase in ruminal acetate concentration and a linear reduction in the *Entodinium* protozoa population in the 33.9 mg/d treatment. Furthermore, this could have implications for energy metabolism and efficiency at low levels and in mixtures of phenolic compounds [44].

These results are consistent with those of the study of [45], who studied the effect on 32 lambs fed with a high-concentrate diet with either palm oil or flaxseed, with and without QCT (2 g/kg TMR for 5 weeks). The study found that changes in the ruminal microbiota occurred only when flaxseed and QCT were combined. However, QCT alone did not affect the ruminal VFA production or microbiota, suggesting that synergism is necessary to influence the ruminal microbiota and that low doses of QCT may be required.

Equally, QCT, CA and LT may exhibit substrate-dependent effects. For example, QCT alone is reported to be rapidly degraded in the rumen, resulting in metabolites without significantly impacting the ruminal gas production, methane emissions or VFA concentrations [46].

It was found that QCT, CA and LT (1 mg/250) mL significantly decreased (*p* < 0.05) after 12 h of incubation in rumen fluid with a 75:25 forage-to-concentrate diet, with over 70% reduction in these bio compounds, observed at 72 h [47]. Diets that extend ruminal retention times can prevent the rapid removal of additives, maintaining their concentrations above the minimum inhibitory levels [48].

Monensin is known to reduce DMI while increasing feed efficiency, and it consistently enhances the proportion of propionate at the expense of acetate, leading to a decreased acetate to propionate (A:P) ratio [49]. However, in our experiment, monensin did not affect the feed efficiency or VFA. This lack of effect could be attributed to the duration of supplementation, which lasted 42 days. The effects of monensin on methanogenesis and ruminal fermentation in ruminants may indeed be time-dependent [50]. Additionally, [51] reported a reduction in the alpha diversity of rumen bacteria following the introduction of monensin, which was gradually restored after 30 days. Therefore, the timing of supplementation may play a crucial role in the observed responses to monensin in our study.

Blood parameters showed no significant adverse effects on energy and liver function, confirming the safety of TDE at 5 g and 10 g/day. Glucose is an indicator of energy status and stress [52,53]. Nutritional deficiencies and liver lesions can significantly alter blood glucose [54]. The levels observed in this study aligned with expected values based on genetics and environment [55], suggesting stable metabolism without stress or deficiencies.

Additionally, decreased blood urea nitrogen (BUN) levels suggest improved protein metabolism. It was observed that the TDE treatments exhibited lower BUN levels, suggesting potential beneficial effects on protein metabolism. The BUN levels are closely associated with the ruminal ammonia levels [56]. Microbial protein production in the rumen is optimized when the balance between available energy (fermentable organic matter) and protein (nitrogen) is ideal [57]. An excess of nitrogen compared with energy, or high nitrogen degradation due to deamination in the rumen, leads to an increase in ruminal ammonia concentration [58] and BUN.

No negative effect on AST or ALT was observed, indicating a safe effect on liver function for the inclusion of TDE at 5 and 10 g/day in the present study. ALT and AST are indicators of liver function. ALT has a longer half-life (40–60 h) than AST (12 h) and is cytosolic, unlike AST which is cytosolic and mitochondrial. After acute injury, AST normalises faster (hours–days) than the longer-lasting ALT (days). After an acute injury, AST normalizes faster (hours–days) than ALT with a longer duration (days); these enzymes are markers of acute as well as subacute hepatocellular damage [59]. 

## 5. Conclusions

Under the conditions in which the experiment was carried out, supplementing lambs with a TDE extract at 10 g/day (TDE10) showed improvements in total tract digestibility of NDF and reduced the ruminal acetate to propionate ratio. Based on the measured blood metabolites, TDE5 and TDE10 did not cause alterations in blood parameters. However, as TDE10 reduced the DM intake, the improvements in digestibility and ruminal fermentation were not reflected in the growth performance improvements. Due to the fact that the lambs that were fed with monensin treatment showed an unusual response, it is not possible to state conclusively that TE is an option to replace monensin in finishing diets.

## Figures and Tables

**Table 1:** Phenolic compounds in aqueous extract of *T. diversifolia*.

Retention Time, min (tR)	Compound	mg/kg
4.00	Caffeic acid	49.80
6.00	Quercetin	1.00
6.20	Luteolin	0.07
6.60	Apigenin	0.30

**Table 2:** Ingredients and chemical composition of the basal diet.

Item	% of DM
Ingredient	
Corn silage	66.18
Corn grain, ground	19.00
Soybean meal	12.50
Urea	1.77
Bicalcium phosphate	0.35
Mineral mixture ^1^	0.20
Bromatological composition	
Crude protein (CP)	14.29
Neutral detergent fibre (NDF)	40.08
Non-structural carbohydrates	35.08
Ether extract (EE)	1.83
Ash	8.02
Total digestible nutrients (TDN)	71.21

^1^ Calcium: 130.0 g (max.); phosphorus: 65.0 g (min.); sodium: 135.0 g; sulphur: 12.0 g; magnesium: 12 g; manganese: 1.050 mg; cobalt: 63 mg; iodine: 63 mg; copper: 1.155 mg; selenium: 18 mg; zinc: 3.080 mg and fluor: 650 mg. Vitamins Premix.

**Table 3:** Effect of *T. diversifolia* extract as an additive on animal performance, DM conversion and feed intake in crossbreed hair lambs supplemented with a forage to concentrate ratio of 66:34.

Parameter	Treatments				SEM ^1^	*p*-Value
	Control	Monensin	5 ^+^	10 ^+^		
	Animal Performance, kg					
Initial BW ^2^	18.95	18.26	18.92	17.98	1.70	0.299
Final BW	27.47	26.33	27.34	25.20	1.96	0.075
ADG ^3^	0.20	0.19	0.20	0.17	0.03	0.223
DM conversion	4.41	4.43	4.46	4.65	0.54	0.952
	Intake kg day^−1^					
Dry matter	0.89 a	0.85 b	0.90 a	0.79 b	0.04	0.000
Crude Protein	0.16 a	0.10 b	0.15 a	0.13 b	0.01	0.000
Neutral detergent fibre	0.38 a	0.30 c	0.37 ab	0.32 b	0.02	0.000
Non-structural carbohydrates	0.38 a	0.28 c	0.36 a	0.32 b	0.01	0.000
Ether extract	0.02	0.01	0.02	0.01	0.00	0.052

^1^ SEM, standard error of means; ^2^ BW, body weight; ^3^ ADG, average daily gain. ^+^ TDE inclusions g day^−1^. Each gram of extract contained 0.0498 mg of caffeic acid, 0.001 mg of quercetin, 0.0007 mg of luteolin and 0.0003 mg of apigenin. Means with different letters have statistical differences, as confirmed by the Fisher test.

**Table 4:** Effect of *T. diversifolia* extracts as an additive on nutrient apparent total tract digestibility and ruminal and VFA in crossbreed hair lambs supplemented with a forage to concentrate ratio of 66:34.

Parameter	Treatments				SEM ^1^	*p*-Value
	Control	Monensin	5 ^+^	10 ^+^		
	Digestibility, g/kg					
Dry matter	72.97	72.85	73.48	73.90	0.94	0.849
Crude Protein	93.40	92.91	93.05	93.12	0.29	0.676
Neutral detergent fibre	55.92 c	58.40 ab	56.93 ab	61.32 a	1.69	0.025
Non-structural carbohydrates	85.74	83.65	85.74	83.90	0.75	0.082
Ether extract	86.68	87.29	87.29	83.63	0.65	0.061
TDN ^2^	61.52	63.88 ab	61.99 c	66.41 a	1.37	0.048
	Volatile Fatty Acid. VFA (%)					
Acetate	76.29 a	77.31 a	76.42 a	67.82 b	1.65	0.042
Propionate	15.72	14.52	16.59	21.07	1.22	0.056
Butyrate	7.99	8.18	7.00	11.12	1.00	0.147
Acetate: Propionate	4.86	5.37	4.70	3.22	0.46	0.106

^1^ SEM, standard error of means; ^2^ TDN, total digestible nutrients. ^+^ TDE inclusions g day^−1^. Each gram of extract contained 0.0498 mg of caffeic acid, 0.001 mg of quercetin, 0.0007 mg of luteolin and 0.0003 mg of apigenin. Means with different letters have statistical differences, as confirmed by the Fisher test.

**Table 5:** Plasma glucose, BUN, AST and ALT concentrations of crossbreed hair lambs supplemented with *T. diversifolia* extract in a diet with a forage to concentrate ratio of 66:34.

Parameter	Treatments				SEM ^1^	*p*-Value
	Control	Monensin	5 ^+^	10 ^+^		
Glucose, mg/dL	81.43	76.80	81.54	81.63	3.79	0.763
BUN, mg/dL	14.45	12.69	12.35	11.49	2.39	0.231
AST, mg/dL	16.18	12.94	12.13	10.93	1.79	0.221
ALT, mg/dL	21.33	33.36	15.84	23.80	8.78	0.569

^1^ SEM, standard error of means. ^+^ TDE inclusions g day^−1^. Each gram of extract contained 0.0498 mg of caffeic acid, 0.001 mg of quercetin, 0.0007 mg of luteolin and 0.0003 mg of apigenin. Sample taken on day 42 of the experiment.

## Data Availability

Data can be made available upon request to the corresponding author.
